# Oleate acid-stimulated HMMR expression by CEBPα is associated with nonalcoholic steatohepatitis and hepatocellular carcinoma

**DOI:** 10.7150/ijbs.49785

**Published:** 2020-08-27

**Authors:** Deyu Zhang, Jiahong Liu, Tian Xie, Qiwei Jiang, Lihua Ding, Jianhua Zhu, Qinong Ye

**Affiliations:** 1 Department of Medical Molecular Biology, Beijing Institute of Biotechnology, Beijing 100850, China.; 2 Department of Oncology, The Fourth Medical Center, PLA General Hospital, Beijing 100048, China.

**Keywords:** non-alcoholic steatohepatitis, hepatocellular carcinoma, bioinformatics, oleate acid, cell cycle, CEBPα

## Abstract

Non-alcoholic steatohepatitis (NASH) is a type of nonalcoholic fatty liver disease and has become a major risk factor for hepatocellular carcinoma (HCC). However, the underlying pathophysiological mechanisms are still elusive. Here, we identify hyaluronan-mediated motility receptor (HMMR) as a critical gene associated with NASH/HCC by combination of bioinformatic analysis and functional experiments. Analysis of differentially expressed genes (DEGs) between normal controls and NASH/HCC identified 5 hub genes (HMMR, UBE2T, TYMS, PTTG1 and GINS2). Based on the common DEGs, analyses of univariate and multivariate Cox regression and the area under the curve (AUC) value of the receiver operating characteristic (ROC) indicate that HMMR is the most significant gene associated with NASH/HCC among five hub genes. Oleate acid (OA), one of fatty acids that induce cellular adipogenesis, stimulates HMMR expression via CCAAT/enhancer-binding protein α (CEBPα). CEBPα increases the expression of HMMR through binding to its promoter. HMMR promotes HCC cell proliferation* in vitro* via activation of G1/S and G2/M checkpoint transitions, concomitant with a marked increase of the positive cell cycle regulators, including cyclin D1, cyclin E, and cyclin B1. Knockdown of HMMR suppresses HCC tumor growth in nude mice. Our study identifies an important role of HMMR in NASH/HCC, and suggests that HMMR may be a useful target for therapy and prognostic prediction of NASH/HCC patients.

## Introduction

Non-alcoholic steatohepatitis (NASH) is a type of nonalcoholic fatty liver disease (NAFLD) characterized by hepatic triglyceride accumulation plus inflammation and hepatocyte injury 1. According to reported data, NAFLD and NASH affect approximately 30% and 5% of the US population 2. NASH is also the main chronic liver disease in the US and Europe, and is increasing worldwide 3. There is mounting epidemiological evidence that it has become an emerging risk factor for hepatocellular carcinoma (HCC) 4. Despite advances in HCC management, it is the second leading cause of cancer death in men and sixth in women worldwide 5. The pathophysiological mechanisms linking NASH to HCC remain poorly understood, and are likely to be multifactorial. These may include dysregulated integrin and Hedgehog signaling, mitochondrial dysfunction, hepatic stellate cell (HSC) activation, and alteration of immune system 6.

Hyaluronan-mediated motility receptor (HMMR), also known as receptor for hyaluronate-mediated motility (RHAMM), is a largely coiled-coil protein that can bind to microtubules and localize to the centrosome 7. HMMR plays an important role in the regulation of spindle assembly in mitotic cells. HMMR expression is cell cycle-regulated with peak expression between late G2 phase and early mitosis. Expression of HMMR is upregulated in a variety of cancers, such as colorectal cancer 8, stomach cancer 9, endometrial cancer 10, prostate cancer 11, and multiple myeloma 12, and its high expression correlates with poor prognosis. In contrast, for some cancers, such as malignant peripheral nerve sheath tumors 13, 14 and seminomas 15, HMMR expression is downregulated and its low expression associates with poor patient survival. Very recently, HMMR was found to be one of the hub genes responsible for prognosis prediction of hepatocellular carcinoma (HCC) by bioinformatic analysis 16-19. However, the clinical significance of HMMR linking normal liver to NASH to HCC and the biological function of HMMR in HCC remains unclear.

In this study, we aim to identify genes linking NASH and HCC through bioinformatics. We show that HMMR may be critical for the development of NASH and further progression to HCC. The accumulation of oleate acid, the most distinctive characteristic of liver tissues with NASH 20, increases HMMR expression through the transcription factor CCAAT enhancer binding protein α (CEBPα). Silencing of HMMR reduces HCC cell proliferation *in vitro* and tumor growth *in vivo*. Mechanistically, HMMR can regulate cell cycle progression in HCC cells.

## Materials and Methods

### Plasmids, cell lines and reagents

FLAG-tagged HMMR expression vector was constructed by inserting PCR amplified HMMR fragment into the pcDNA3 vector (Invitrogen) linked with FLAG tag at the amino terminus. The HMMR promoter luciferase reporters were made by inserting PCR-amplified HMMR promoter fragments into the pGL4-Basic vector (Promega). HepG2 and MHCC-97H liver cancer cell lines were purchased from the American Type Culture Collection (Manassas, VA, USA). Small interfering RNAs (siRNAs) were synthesized by JTS scientific or GemmaPharma. The cDNA target sequences of siRNAs and/or short hairpin RNAs (shRNAs) for HMMR and CEBPα were listed in Table S1. Stable cell lines overexpressing HMMR shRNA were established by lentiviral transduction using pSIH-H1-Puro carrying HMMR shRNA. Anti-cyclin D1, anti-cyclin E, anti-cyclin B1 and anti-CEBPα were purchased from Santa Cruz Biotechnology; Anti-HMMR was from Proteintech.

### Data collection

The GSE89632 and GSE126848 datasets were downloaded from the Gene Expression Omnibus (GEO, https://www.ncbi.nlm.nih.gov/geo/) database. The GSE89632 dataset contains gene expression profiles of 19 patients with NASH and 24 healthy controls 21. The GSE126848 dataset contains gene expression profiles of 16 patients with NASH and 14 healthy controls 22. In addition, mRNA expression profiles and clinical data from 366 HCC and 50 normal control samples were obtained from The Cancer Genome Atlas (TCGA, http://cancergenome.nih.gov) database (TCGA-LIHC) 23. The 50 normal control samples had matched tumor tissues.

### Identification of differentially expressed genes (DEGs) in NASH and HCC

To investigate differentially expressed genes (DEGs) in each GEO dataset and TCGA-LIHC dataset, we used the limma R package 24. By controlling the false discovery rate (FDR), we define DEGs as genes with adj. *P*-value <0.05 and |log_2_ fold change (FC)|>1. The intersecting DEGs of NASH and HCC were used for further analysis.

### Functional and pathway enrichment analysis

Functional and pathway enrichment analyses were performed for DEGs using the clusterProfiler R package 25, including Gene Ontology (GO) terms and Kyoto Encyclopedia of Genes and Genomes (KEGG) pathway enrichment analyses 26. GO terms and KEGG pathways with an adj. *P*-value <0.05 were considered significant.

### Protein-protein interaction (PPI) network construction, module analysis and hub gene identification

The PPI network was retrieved from the Search Tool for the Retrieval of Interacting Genes (STRING, http://www.string-db.org/). MCODE plugin in Cytoscape was used to identify the most significant module in the network based on the graph-theoretic clustering algorithm 27. To identify hub genes in the PPI network, we use the cytoHubba plugin for Cytoscape 28. Five analysis methods, namely Degree, Edge Percolated Component (EPC), Maximum Neighborhood Component (MNC), Density of Maximum Neighborhood Component (DMNC), and Maximal Clique Centrality (MCC), were used.

### Survival analysis of hub genes based on the TCGA-LIHC dataset

After excluding HCC patients without survival data, the survival R package was used to conduct survival analyses based on hub gene expression and overall survival (OS) in 365 HCC patients from TCGA-LIHC. Additionally, the online database Gene Expression Profiling Interactive Analysis (GEPIA) (http://gepia.cancer-pku.cn/) was used for disease-free survival (DFS) analysis of hub genes in HCC patients from TCGA-LIHC. Log-rank *P* value <0.05 is the threshold of statistical significance.

### HCC-specific prognostic model construction and assessment

Univariate Cox proportional hazards regression analyses were used to identify individual common DEGs between NASH/HCC and adjacent normal tissues that affect the OS of 365 HCC patients from the TCGA-LIHC dataset. Multivariate Cox regression analysis was used to establish a linear joint risk score of gene expression level (exp) using regression coefficient β. The risk score for each sample was calculated as follows: Risk score = ∑βi×expRNAi 29. For survival analysis, the samples were divided into high- and low-risk groups based on the median or the best cutoff of risk scores. Subsequently, univariate and multivariate Cox regression analysis were performed to identify that the predictive effect of the prognostic model is independent of clinical factors. The prognostic model was assessed by the area under the curve (AUC) value of the receiver operating characteristic (ROC) curve, which was calculated using the survival ROC package of R. Finally, nomogram that integrates risk scores and other clinical factors was constructed to forecast the likelihood of 1-, 3-, 5-OS using the rms R package, and *P* value <0.05 is the threshold of statistical significance.

### Cell culture and Oil Red O staining

Cells were maintained in Dulbecco's modified Eagle's medium (DMEM) supplemented with 10% fetal bovine serum at 37 °C under a humidified atmosphere of 5% carbon dioxide. Cells were plated in 6-well plates at a density of 60% confluency. The next day, cells were treated with 25 μM/50 μM oleate acid or DMSO (as a control), respectively. After treatment for 24 h, cells were stained with Oil Red O and counterstained with hematoxylin 30.

### Transient Transfections

For plasmid transfection, cells were seeded to 70-90% confluency at the time of transfection. The cells were transfected with the indicated plasmids using Vigofect according to the manufacturer's protocol (Vigorous Biotechnology). The transfected cells were collected after 24-48 h. For siRNA transfection, Lipofectamine RNAiMAX reagent was used according to the manufacturer's instructions (Invitrogen).

### Reverse-transcription quantitative real-time polymerase chain reaction (RT-qPCR)

Total RNA was extracted from cultured cells and reverse-transcribed to cDNA using the RNeasy Mini kit (Qiagen) according to the manufacturer's protocol. Expression of mRNAs was determined by quantitative real-time polymerase chain reaction (qPCR) using SYBR Premix Ex Taq Master Mix (Takara). The relative expression was calculated by the comparative Ct method. The sequences of the primers used for RT-qPCR analysis are presented (Table S2).

### Cell proliferation and colony formation assays

Cell proliferation was assessed by a CCK-8 Kit according to the manufacturer's instructions (Dojindo). For colony formation assay, transfected cells were seeded in 6-well plates at 3000 cells per well. Two weeks later, colonies were fixed with 4% paraformaldehyde and stained with 0.5% crystal violet for 30 min. The number of colonies with diameters of more than 1.5 mm was counted.

### Luciferase reporter assay

Cells seeded into 24-well plates were co-transfected with myc-CEBPα, the luciferase reporter with either wild-type or mutant HMMR promoter, and β-galactosidase reporter (internal control). Forty eight hours later, cells were harvested and analyzed for luciferase and β-galactosidase activities according to the manufacture's instruction (Promega). All transfection experiments were performed in triplicates and repeated 3 times.

### Chromatin immunoprecipitation

ChIP assay was performed using the Magna ChIP Assay Kit (Millipore) according to the manufacturer's instructions. The collected DNA fragments were quantified by qPCR with listed primers (Table S2).

### Cell cycle analysis

Cell cycle analysis was carried out using flow cytometry. Briefly, cells were fixed in 70% ethanol for more than 12 h. After washing with PBS, fixed cells were incubated with RNase A (0.2 mg/mL) in PBS. Propidium iodide was then added to the cell suspension. Samples were analyzed by a FACSCalibur Flow Cytometer (Becton Dickinson).

### Statistical analysis

All the experiments were performed in triplicate and repeated 3 times. Data are expressed as mean ± standard deviation (SD), and were analyzed using SPSS 17.0 or R software. Statistical significance in cell line experiments was assessed by a two-tailed Student's *t* test. *P* < 0.05 was considered to be statistically significant.

## Results

### Identification of commonly regulated differentially expressed genes between NASH/HCC and normal liver tissues

**Figure 1:**
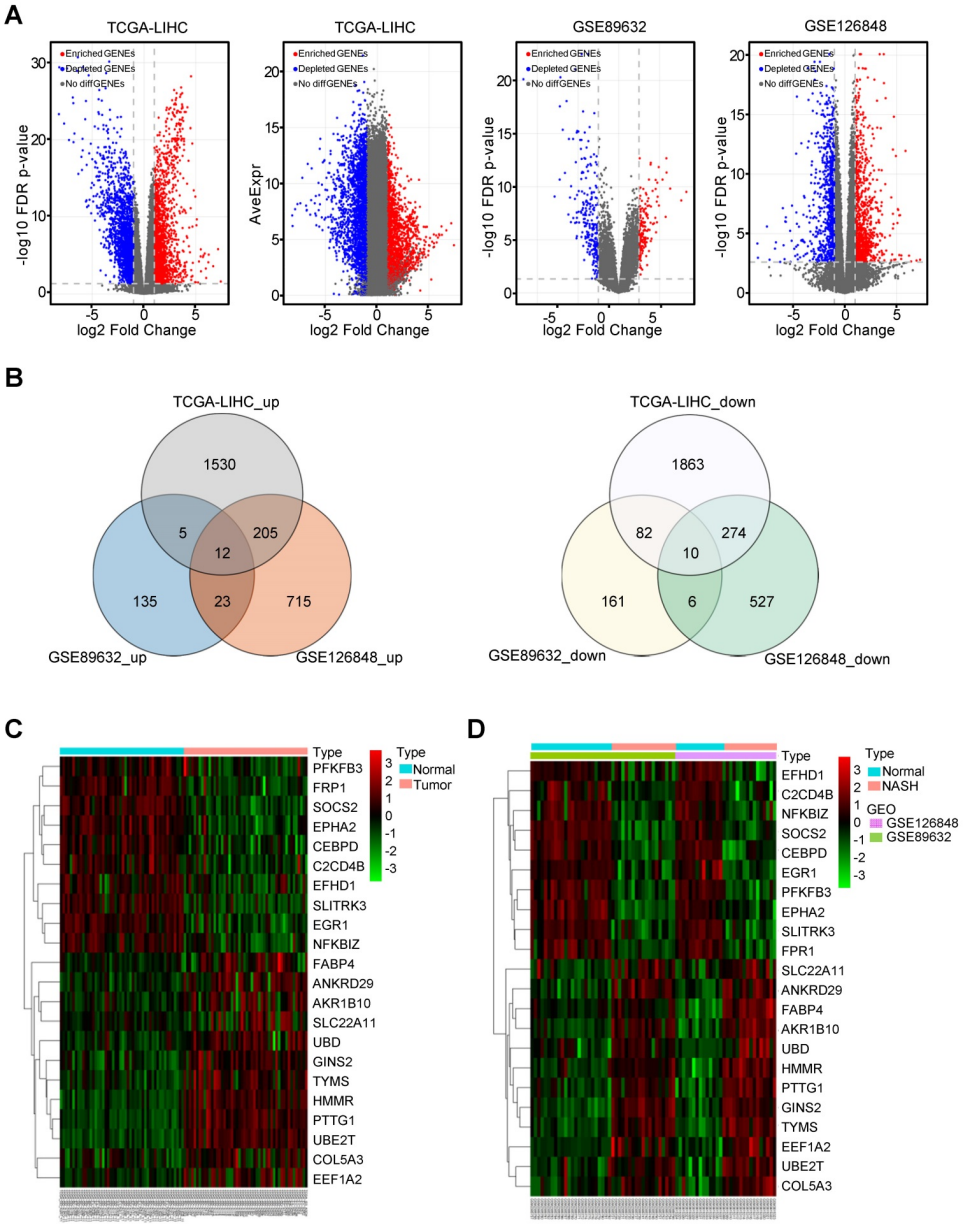
** The common differentially expressed genes (DEGs) between normal controls and NASH/HCC.** (A) The volcano plot showing DEGs in 366 HCC samples from TCGA-LIHC dataset, 43 samples from GSE89632 and 30 samples from GSE126848 dataset, respectively. (B) The common differentially up/down-expressed genes based on GSE89632, GSE126848 and TCGA-LIHC datasets. (C and D) The heatmaps illustrating the expression of common DEGs based on TCGA-LIHC (C), GSE89632 and GSE126848 (D) datasets.

A total of 50 paired HCC and adjacent normal tissues from the TCGA-LIHC dataset were analysed. The clinicopathological features were shown in Table S3. In this dataset, 1530 upregulated and 1863 downregulated differentially expressed genes (DEGs) were identified in HCC tissues compared with non-tumor tissues. In the GSE89632 dataset, 135 upregulated and 161 downregulated DEGs were identified in NASH patients compared with healthy controls. In the dataset GSE126848, 715 upregulated and 527 downregulated DEGs were identified in NASH patients compared with healthy controls. The volcano plot illustrating the gene expression profile of this dataset was shown in Figure 1A. There were 12 common upregulated DEGs and 10 common downregulated DEGs between NASH/HCC and adjacent normal tissues (Figure 1B). Seven genes out of the 22 common DEGs, such as FABP4, AKR1B10, and UBD, have been demonstrated to be the critical genes related to both NASH and HCC 31-36. Some of the other 15 genes have been reported to be associated with only NASH or HCC. In addition, the heatmap illustrated the expression of common DEGs in HCC tissues or non-tumor tissues from the TCGA-LIHC (Figure 1C). Another heatmap illustrated the expression of common DEGs in NASH tissues or normal liver tissues (Figure 1D).

### PPI network construction and hub gene identification

**Figure 2:**
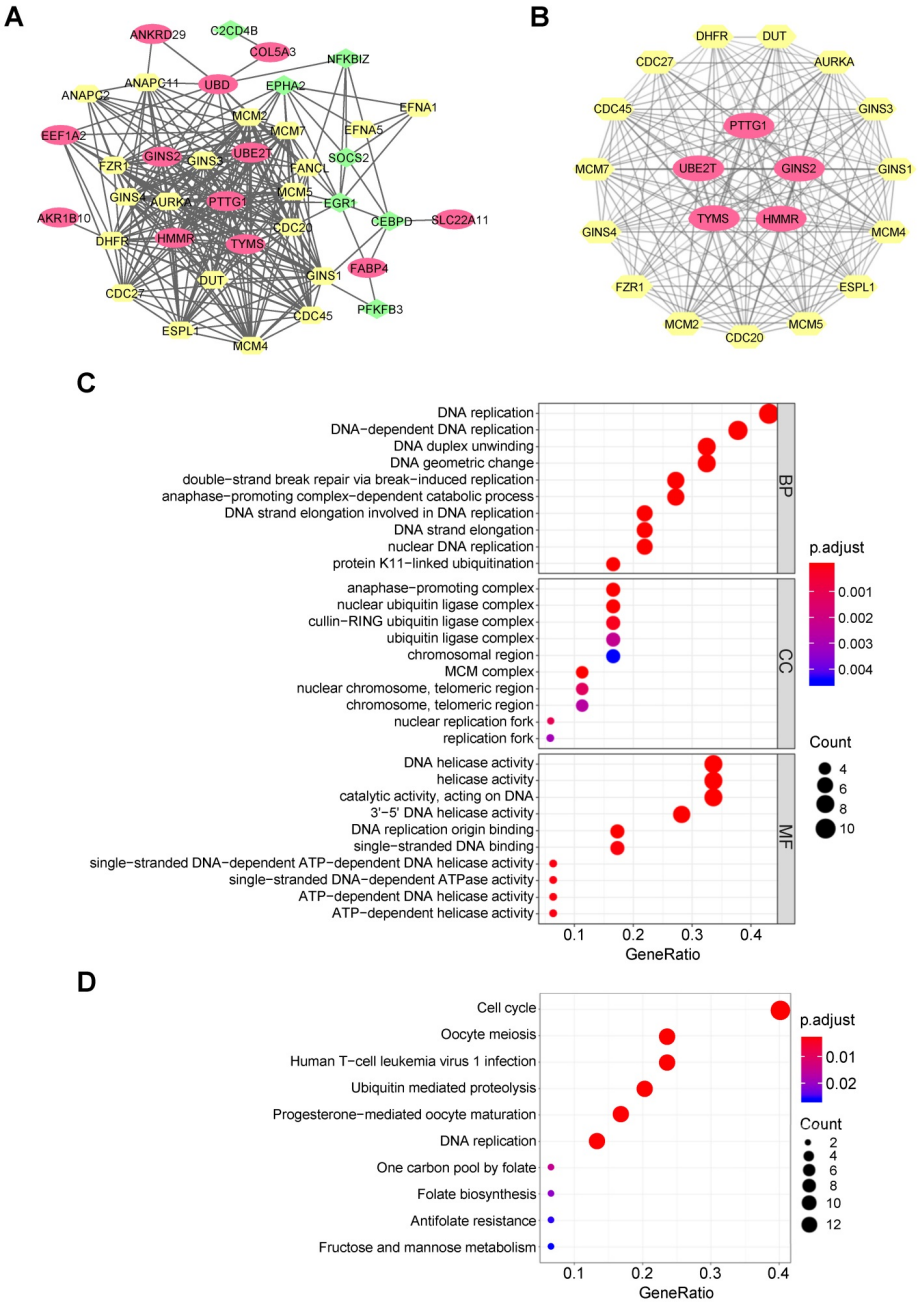
** Functional enrichment analysis based on the DEGs.** (A) Entire PPI network analysis of the DEGs and their direct interactors. (B) Identification of the most significant module and hub genes of the DEGs. (C) The top 10 changes in BP/MF/CC of GO analysis based on the DEGs and their direct interactors. (D) Top 10 enriched KEGG pathways based on the DEGs and their direct interactors.

A PPI network for 22 common DEGs was built by the STRING online database, and 20 most credible direct interactors were joined in analysis (Figure 2A). The network shows the interconnectedness of 22 common DEGs between NASH/HCC, with upregulated ones being red and downregulated ones being green. There were several PPI prediction clusters analyzed by MCODE plugin in Cytoscape. Finally, we extracted the most significant module, which included five upregulated DEGs and 15 interactors (Figure 2B). These five upregulated DEGs also were identified as top five hub genes among DEGs by algorithms, MCC and DMMC (Table S4). Thus, the five genes, namely HMMR, ubiquitin conjugating enzyme E2 T (UBE2T), thymidylate synthetase (TYMS), pituitary tumor transforming gene 1 (PTTG1), and GINS complex subunit 2 (GINS2), were chosen for further analysis.

### Functional and pathway enrichment analysis

For 22 common DEGs and the top 20 direct interactors, GO terms showed that changes in biological process included significant enrichment of DNA replication, mitosis associated catabolic process, etc. (Figure 2C). Changes in molecular function (MF) included significant enrichment of DNA helicase activity, DNA replication origin binding, ribonucleotide binding, single-stranded DNA-dependent ATPase activity, etc. Changes in cellular component (CC) included significant enrichment of genes related to the cell nucleus. KEGG pathway enrichment analysis demonstrated that cell cycle, meiosis, DNA replication and folate biosynthesis were significantly enriched (Figure 2D). The enrichment of cell cycle was consistent with previously reported researches on the five hub genes. HMMR was shown to bind to microtubule during mitotic spindle formation, suggesting its function in cell division 37. UBE2T knockdown induces G1/S cell cycle arrest in HCC cells 38. Silencing of PTTG1 inhibits cell proliferation and inhibits cyclin D1 expression in cholangiocarcinoma cells 39. TYMS is an essential rate-limiting enzyme in the nucleotide metabolism, and is involved in DNA synthesis 40. GINS2 knockdown also inhibits cell viability and induces cell cycle arrest in pancreatic cancer cells 41. Thus, abnormal cell cycle may be the intrinsic related mechanism of NASH and HCC. Taken together, both GO terms and KEGG analysis indicated that some common DEGs might play an important role in cell cycle regulation in HCC cells and might have a role for NASH to HCC.

### The validation of five hub genes in TCGA-LIHC dataset

**Figure 3:**
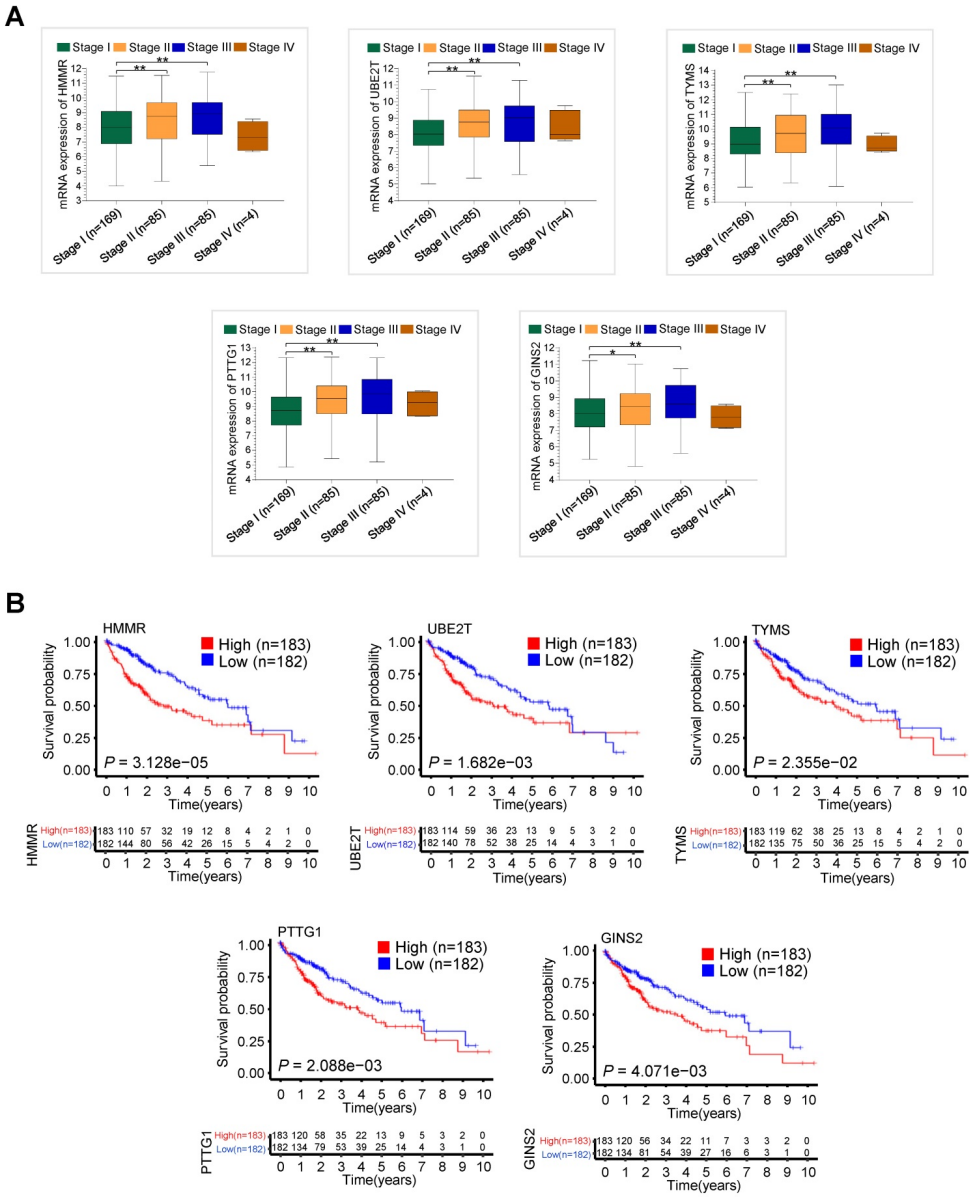
** The validation of clinical significance of five hub gene expression in HCC patients from TCGA-LIHC dataset.** (A) The relationship between five hub gene mRNA expression and clinical stages in 343 HCC patients from TCGA-LIHC dataset (*P < 0.05 versus stage I, **P < 0.01 versus stage I). (B) The association between five hub gene expression and overall survival in 365 HCC patients from TCGA-LIHC dataset.

We further explored associations between the hub gene expression and clinical stage in 343 HCC patients with clinical information from TCGA-LIHC dataset. The mRNA expression levels of HMMR, UBE2T, TYMS, PTTG1 and GINS2 significantly increased in those with advanced clinical stage (Figure 3A). Notably, the expression levels decreased in stage IV, which might be due to the small sample size. Moreover, high mRNA expression of the five hub genes, especially HMMR, was significantly associated with shorter overall survival (OS) in 365 HCC patients with survival information from TCGA-LIHC dataset (Figure 3B). High mRNA expression of the five hub genes was also correlated with shorter disease free survival (DFS) (Figure S1).

### Identification of prognostic signature

**Figure 4:**
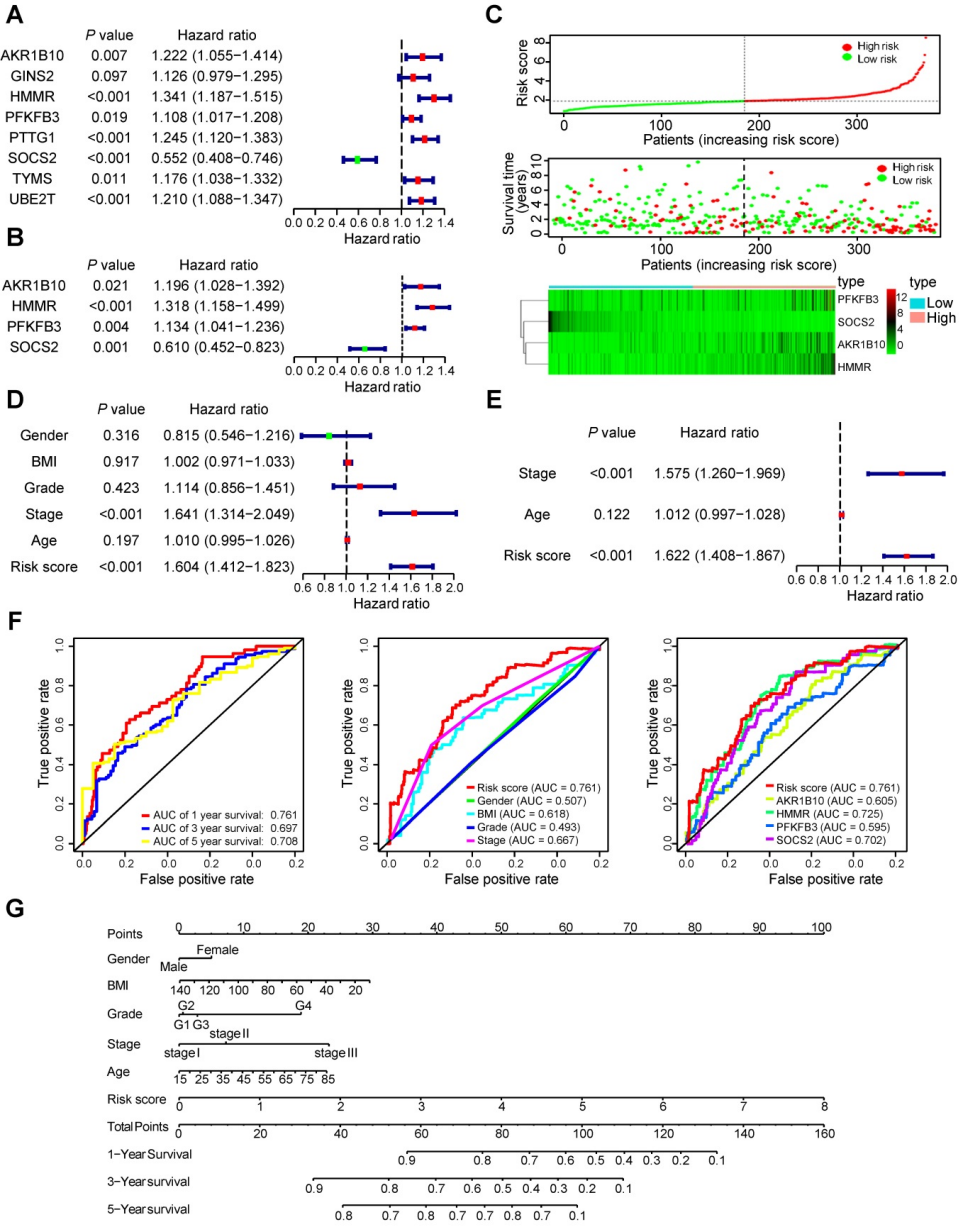
** The 4-mRNA prognostic signature and the comprehensive nomogram constructed for HCC patients.** (A and B) The forest plot exhibited genes significantly correlated with overall survival based on univariate (A) and multivariate (B) Cox regression analysis of 22 common DEGs. (C) The distribution of the risk score, survival status and gene expression of the 4-mRNA prognostic signature. (D and E) The forest plot exhibited prognostic factors significantly correlated with overall survival based on univariate (D) and multivariate (E) Cox regression analysis of risk score and other clinical features. (F) ROC curve was plotted for the prognostic model with 1-, 3- and 5-year overall survival, clinical features, and individual HMMR, AKR1B10, PFKFB3 or SOCS2 in HCC patients. (G) The comprehensive nomogram for 1-, 3- and 5-year overall survival prediction of HCC patients.

To determine the association between common DEGs and patients' outcomes, 22 common DEGs were firstly submitted to univariate Cox proportional hazards regression. Eight genes, involving all five hub genes, were identified to have a significant prognostic value (Figure 4A). Then, multivariate Cox proportional hazards regression analysis screened out four genes: HMMR, aldo-keto reductase family 1 member B10 (AKR1B10), 6-phosphofructo-2-kinase/fructose-2,6-bisphosphatases (PFKFB3), and suppressor of cytokine signaling 2 (SOCS2) (Figure 4B). Four-mRNA based prognostic signature was constructed and the risk-score formula used to calculate the risk score was as follows: (0.276*HMMR + 0.179*AKR1B10 + 0.126*PFKFB3 - 0.495*SOCS2). The concordance index of this prognostic model was 0.692, indicating a certain predictive effect. According to the median value of the prognostic risk score, 365 HCC patients from the TCGA-LIHC dataset were divided into low- and high-risk groups. The distribution of the risk score along with the corresponding OS data and the expression level of three genes in the prognostic model were plotted (Figure 4C). As depicted in the picture, patients with higher risk scores tended to experience a shorter OS time and higher death rate.

### Prognostic signature validation and nomogram construction

We conducted the univariate Cox proportional hazards regression analysis to screen significant clinical features for prognosis, including age, gender, body mass index (BMI, weight in kilograms divided by the square of height in metres), grade, pathologic stage and the risk score of prognostic model were included. Only pathologic stage and the risk score of prognostic model had an effect on prognosis of HCC patients (Figure 4D). To determine whether the 4-mRNA-based prognostic signature is independent prognostic factor for HCC patients, the multivariate Cox proportional hazards regression analysis was performed using risk score and other clinical features. Pathologic stage and risk score maintained a significant and independent factor for prognosis prediction (*P* < 0.001) (Figure 4E). Additionally, the area under the curve (AUC) value of prognostic model was 0.761, 0.697 and 0.708 in 1-, 3-, 5-year survival prediction, respectively (Figure 4F). According to 1-year survival prediction, the AUC value of prognostic model was superior to other clinical factors. The AUC value of HMMR alone was 0.725, and was an alternative to the prediction model with the four genes, HMMR, AKR1B10, PFKFB3, and SOCS2 (0.761). The comprehensive nomogram was constructed for individualized prediction of 1-, 3-, and 5-year OS that intergrated prognostic features (age, gender, BMI, grade, pathologic stage and risk score) (Figure 4G).

### Oleate acid elevates the expression of HMMR via CEBPα

**Figure 5:**
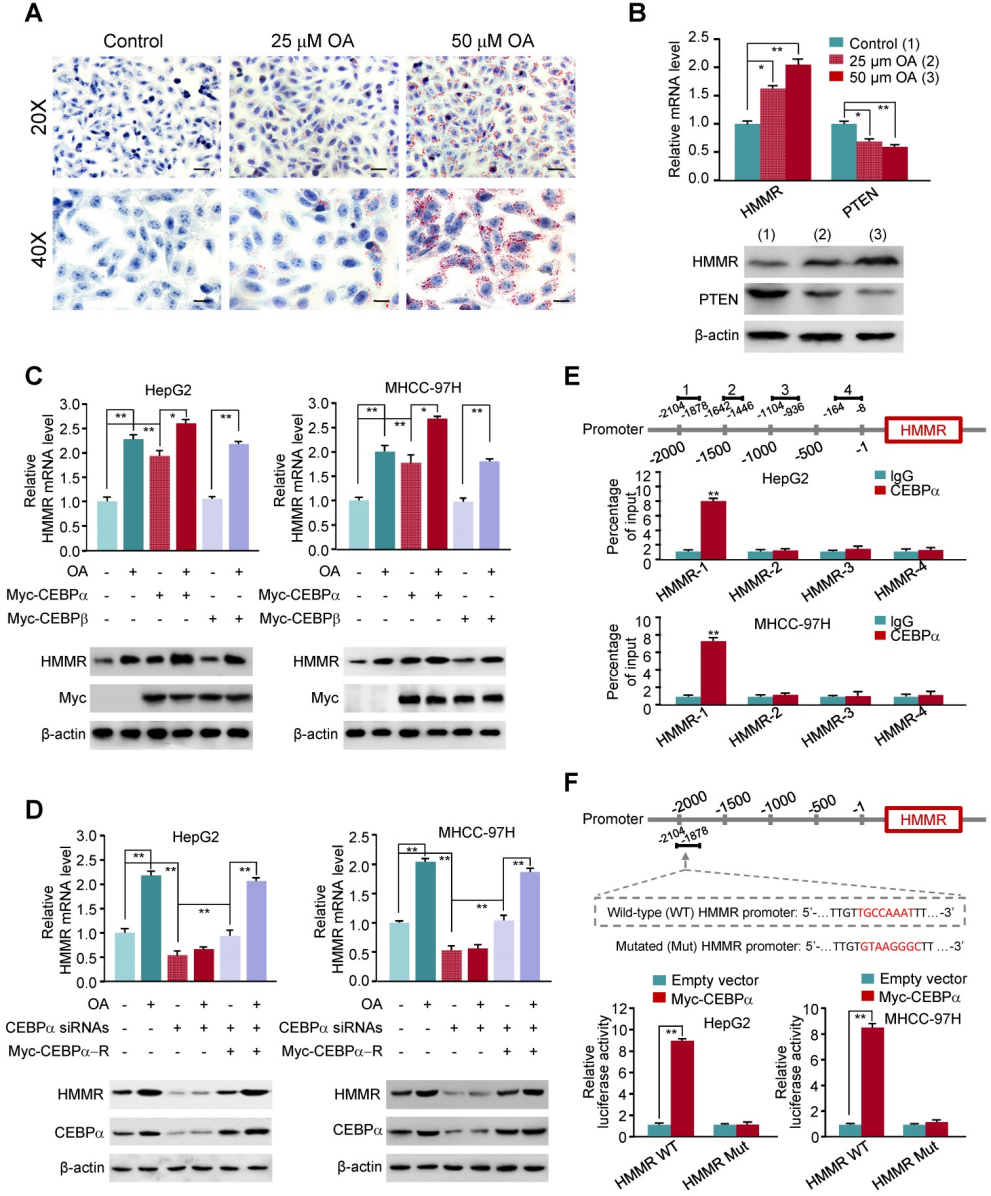
** Oleate acid induces HMMR expression via CEBPα.** (A) Elevated fat storage in HepG2 cells treated with 25 μM and 50 μM oleate acid. Cells were stained with Oil Red O and counterstained with hematoxylin. Scale bar, 50 μm (20X) and 25 μm (40X). (B) RT-qPCR and immunoblot analysis of HepG2 cells treated as in (A). RT-qPCR was used for examination of mRNA expression of HMMR and PTEN, and representative immunoblot for their protein expression. β-actin was used as a loading control. (C) RT-qPCR and immunoblot analysis of HepG2 and MHCC-97H cells transfected with empty vector or Myc-tagged CEBPα or CEBPβ and treated with 50 μM oleate acid. (D) RT-qPCR and immunoblot analysis of HepG2 and MHCC-97H cells transfected with control siRNA, CEBPα siRNAs or CEBPα siRNAs plus siRNA-resistant CEBPα plasmid (Myc-CEBPα-R) and treated with 50 μM oleate acid. (E) ChIP analysis of CEBPα occupancy on HMMR promoter in HepG2 cells. IgG, normal serum. The different number represents the regions upstream of the transcriptional start site (-1) of HMMR promoter. (F) Luciferase reporter assays of HepG2 and MHCC-97H cells transfected with wild-type or mutated HMMR reporters and Myc-CEBPα. A schematic diagram of the HMMR promoter reporter constructs is shown. Red fonts indicate the putative CEBPα-binding site in human wild-type HMMR promoter. Mutations are introduced into the HMMR promoter. Data shown are mean ± SD of triplicate measurements that have been repeated 3 times with similar results (B-F) (*P < 0.05, **P < 0.01).

As the AUC value of ROC analysis for the hub gene HMMR alone is similar to that of 4-mRNA-based prognostic signature, we explored the mechanism of elevated HMMR expression in NASH and HCC. NASH is characterized by accumulation of fat in liver cells. Oleate acid (OA), one of fatty acids that induce cellular adipogenesis, can promote the proliferation of HCC cells 42, 43. OA-induced lipid accumulation in HepG2 liver cancer cells is a well-established model for the investigation of hepatic steatosis 20. As expected, Oil Red O staining showed that HepG2 cells treated with OA exhibited elevated intracellular lipid storage compared to the control cells (Figure 5A). Consistent with previous report (25), OA reduced the expression of phosphatase and tensin homolog (PTEN) (Figure 5B), a vital tumor suppressor gene in hepatocellular carcinoma. Importantly, OA markedly stimulated HMMR expression at the transcriptional and translational levels.

To investigate the mechanism underlying OA-induced HMMR expression, we predicted transcription factors based on the HMMR promoter by TFBIND. Among them, CEBPα is a key transcription factor associated with lipid metabolism, and was reported to be induced by OA 44. Indeed, CEBPα overexpression increased HMMR mRNA and protein expression in HepG2 and MHCC-97H liver cancer cells, and OA further promoted CEBPα-stimulated HMMR expression (Figure 5C). As a control, CEBPβ did not alter the expression of HMMR. In contrast, in HepG2 and MHCC-97H cells, knockdown of CEBPα reduced HMMR mRNA and protein expression, and almost abrogated OA-induced HMMR expression (Figure 5D), indicating CEBPα-dependent HMMR induction by OA. ChIP assay showed that CEBPα was recruited to a region approximately 700-bp upstream of HMMR transcriptional start site, but not the other regions (Figure 5E). Luciferase reporter assays demonstrated that CEBPα increased the activity of wild-type HMMR promoter reporter containing putative CEBPα binding site, but not the reporter in which the putative binding site for CEBPα was mutated, in HepG2 and MHCC-97H cells (Figure 5F). These data suggest that CEBPα promotes HMMR gene transcription through binding to its promoter.

### HMMR promotes HCC cell proliferation

**Figure 6:**
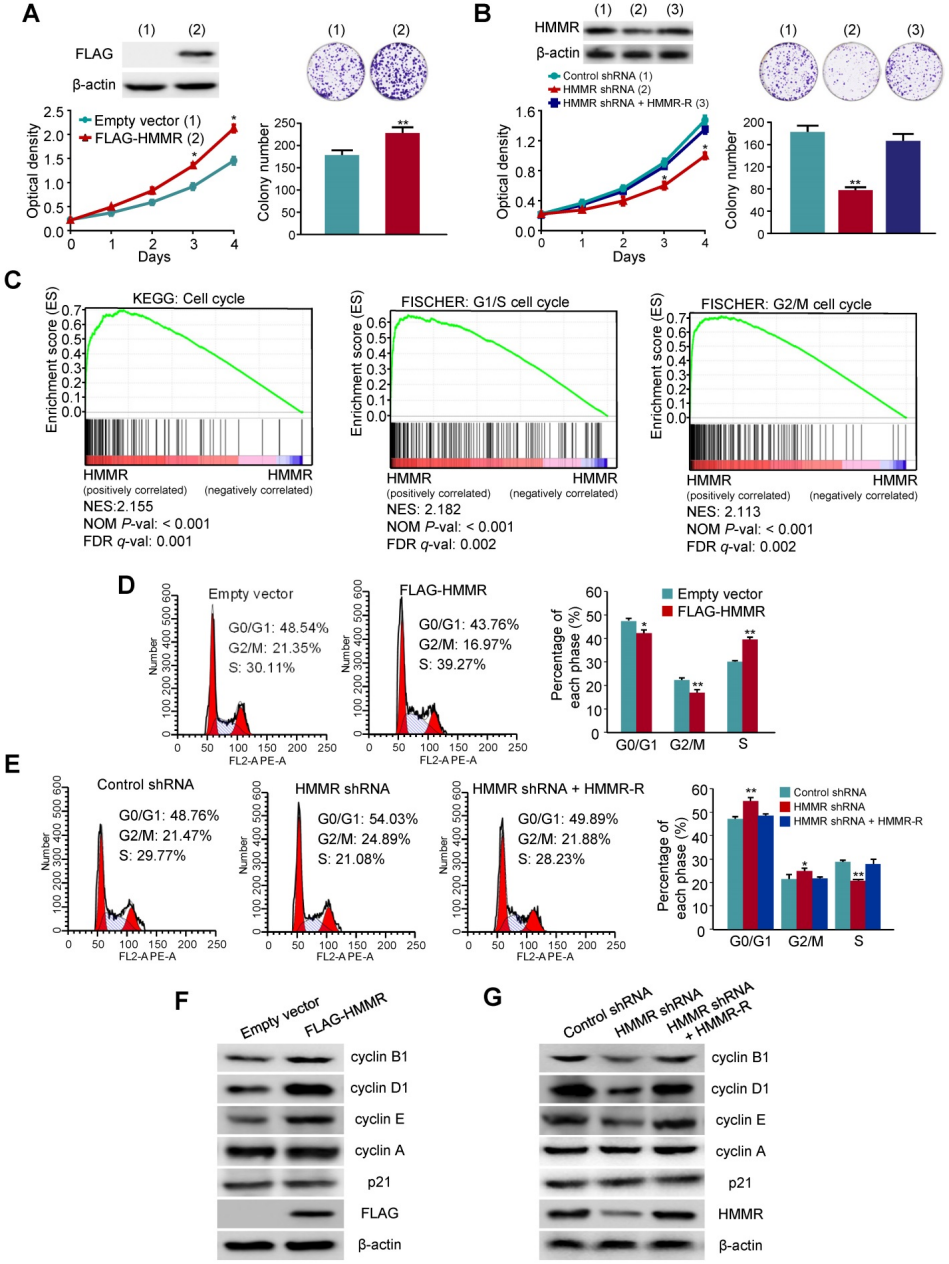
** HMMR promotes HepG2 cell proliferation through activating the G1/S and G2/M transitions.** (A) HepG2 cells transfected with FLAG-tagged HMMR or empty vector were grown in regular medium and harvested at the indicated times. Cell number and colony formation were detected. Immunoblot showed the expression of FLAG-HMMR. (B) HepG2 cells infected with control shRNA, HMMR shRNA or HMMR shRNA plus siRNA-resistant HMMR (HMMR-R) were cultured and analyzed as in (A). Immunoblot showed HMMR expression. (C) GSEA was conducted to predict the potential mechanism of HMMR promoting HepG2 cell proliferation. (D) Flow cytometry analysis of cell cycle in HepG2 cells transfected with FLAG-HMMR or empty vector. (E) Flow cytometry analysis of cell cycle in HepG2 cells infected with HMMR shRNA or HMMR shRNA plus HMMR-R. The image displayed is one of the representative results. Data shown are mean ± SD of triplicate measurements that have been repeated 3 times with similar results (A, B, D and E) (*P < 0.05, **P < 0.01 versus corresponding control). (F) Representative immunoblot of HepG2 cells transfected with FLAG-tagged HMMR or empty vector. (G) Representative immunoblot of HepG2 cells infected with control shRNA, HMMR shRNA or HMMR shRNA plus HMMR-R.

Next, we explored whether HMMR plays a role in HCC cell proliferation. We first investigated the effect of HMMR overexpression on anchorage-dependent growth of HCC cells. HepG2 cells transfected with FLAG-tagged HMMR grew much faster than those transfected with empty vector (Figure 6A). Moreover, colony formation assays revealed that colony number and colony size were larger in HMMR-overexpressing HepG2 cells than those in empty vector-containing cells. In contrast, HepG2 cells transfected with HMMR shRNA grew more slowly than those transfected with control shRNA (Figure 6B). Reexpression of HMMR in the HMMR knockdown cells rescued this effect. Knockdown of HMMR with HMMR shRNA in HepG2 cells decreased the colony number and size. Again, reexpression of HMMR in the HMMR knockdown cells rescued this effect. Similar results were obtained in MHCC-97H cells infected with HMMR-expressing or HMMR shRNA plasmids (Figure S2A and S2B). These results reveal that HMMR increases the proliferation and colony formation of HCC cells.

### HMMR activates the G1/S and G2/M transitions in HCC cells

To elucidate the mechanism by which HMMR promotes HCC cell proliferation, we analyzed enriched pathways by GSEA with 366 HCC samples from the TCGA-LIHC dataset. Interesting, the cell cycle pathway was significantly enriched (Figure 6C). Hence, we investigated the effect of HMMR on cell cycle distribution by flow cytometry analysis. Compared with the control cells, overexpression of HMMR in HepG2 cells resulted in a reduction in the proportion of cells in G0/G1 phase (from 48.54% to 43.76%) and G2/M phase (from 21.35% to 16.97%) but an increase in the proportion of cells in S phase (from 30.11% to 39.27%) (Figure 6D). In contrast, knockdown of HMMR in HepG2 cells significantly increased the proportion of cells in both G0/G1 (48.76% to 54.03%) and G2/M phase (from 21.47% to 24.89%), accompanied by decreased proportion of cells in S phase (29.77% to 21.08%) (Figure 6E). Reexpression of HMMR in the knockdown cells recued these effects. Similar results were obtained in MHCC-97H cells (Figure S2C and S2D). These data suggest that HMMR activates both the G1/S and the G2/M transitions in HCC cells.

### HMMR regulates the expression of G1 and G2 phase-related proteins in HCC cells

Since HMMR regulates cell cycle distribution, we examined the expression of several important cell cycle-related proteins in HMMR knockdown or overexpressing HCC cells. Overexpression of HMMR in HepG2 cells and MHCC-97H cells increased the expression of the G1/S-phase markers cyclin D1 and cyclin E, as well as the G2/M-phase marker cyclin B1 (Figure 6F and Figure S2E). However, the expression of cyclin A and the cell cycle inhibitor p21 was not changed in HMMR- overexpressing cells. On the contrary, knockdown of HMMR in HepG2 cells and MHCC-97H decreased the expression of cyclin D1, cyclin E and cyclin B1 (Figure 6G and Figure S2F). Reexpression of HMMR in the HMMR knockdown cells rescued this effect. Consistent with the HMMR overexpression results, HMMR knockdown did not alter the expression of cyclin A and p21.

### Knockdown of HMMR suppresses HCC tumor growth in nude mice

**Figure 7:**
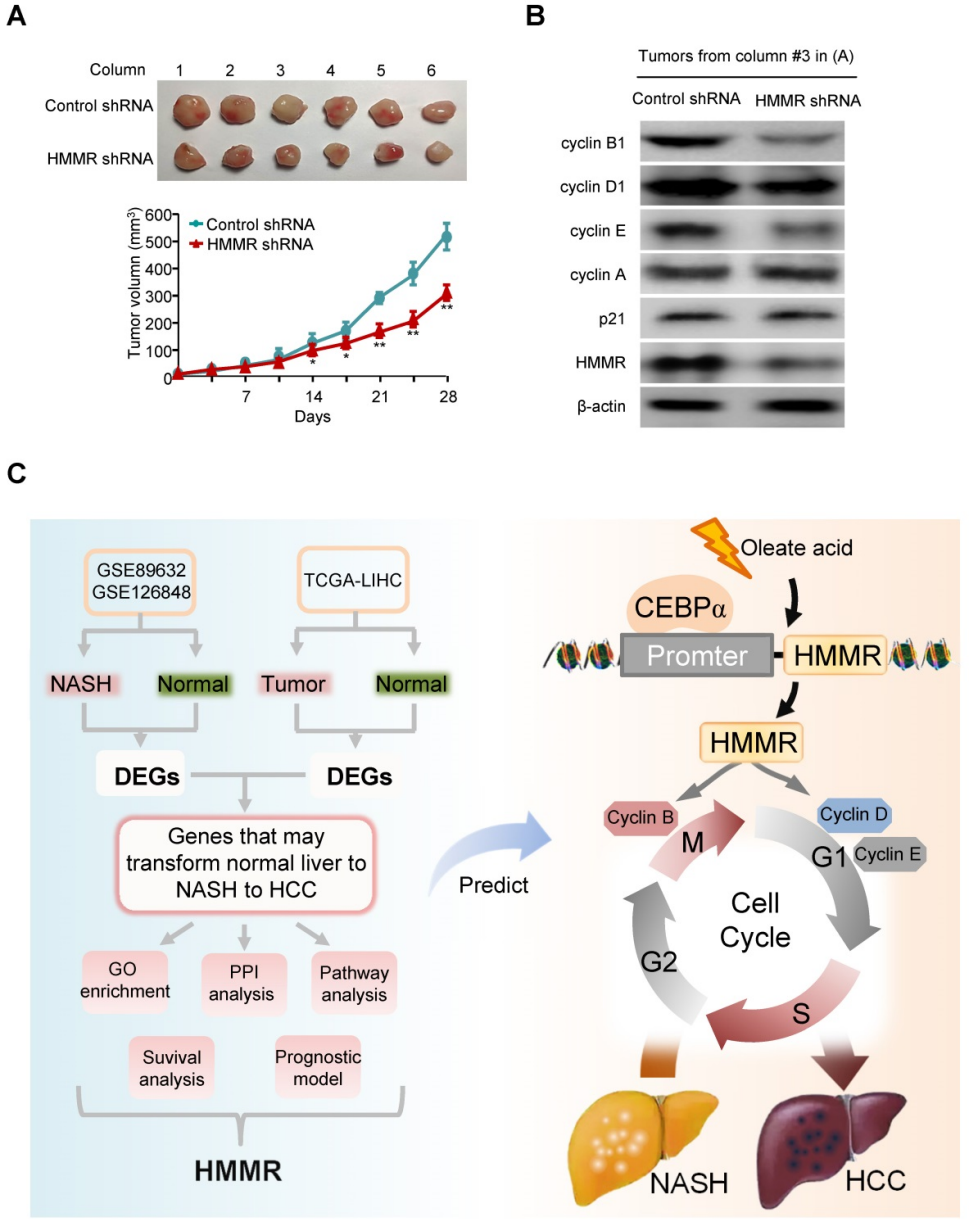
** Knockdown of HMMR suppresses HCC tumor growth in nude mice.** (A) HepG2 cells stably infected with HMMR shRNA or control shRNA were injected subcutaneously in the dorsal of nude mice. The volume of the tumors was examined at the indicated times. Data are shown as mean ± SD (n = 6) (*P<0.05, **P<0.01 versus control shRNA at the corresponding times). (B) Immunoblot analysis of representative tumor tissues from (A). (C) Proposed model for the function of HMMR during the development of normal liver to NASH to HCC. Oleate acid induces the expression of HMMR in a CEBPα-dependent manner. HMMR increases expression of cyclin D1, cyclin E and cyclin B1, thus activating G1/S and G2/M checkpoint transitions.

Next, the effect of HMMR knockdown on HCC tumor growth in nude mice was investigated. HepG2 cells stably infected with HMMR shRNA lentivirus or empty vector were injected subcutaneously in the dorsal of each nude mouse. Compared with the control groups, knockdown of HMMR significantly suppressed HCC tumor growth in nude mouse (Figure 7A). As expected, the HepG2 tumors in mice inoculated with HMMR shRNA showed decreased expression of HMMR, cyclin D1, cyclin E, and cyclin B1 (Figure 7B).

## Discussion

In this study, 12 common upregulated and 10 common downregulated DEGs are identified in NASH and HCC when compared with normal controls. Five genes, including HMMR, UBE2T, TYMS, PTTG1 and GINS2 are identified as hub genes. All five hub genes are found to be independent adverse prognostic biomarkers for OS and DFS. Univariate and multivariate Cox regression analysis and prognostic model analysis reveal that HMMR is the most significant gene associated with NASH/HCC among five hub genes. Furthermore, HMMR promotes HCC tumor growth by activation of cell cycle progression, accompanied by changes in expression of cell cycle regulators (Figure 7C). These findings indicate that HMMR may play an important role in the development of NASH and further progression to HCC.

NASH has become a major carcinogenic factor for HCC, due to the increase in obesity worldwide 1, 45. There is growing literature suggesting that various aspects contribute to the development of this prevalent and serious chronic disease, such as molecular events, immune status, biochemical reaction, and genetic function 46-48. However, the pathophysiologic development of NASH and subsequent progression to HCC is still largely unclear 49. Our study identifies genetic and molecular events, which may be involved in the process from normal liver to NASH to HCC through bioinformatic analysis. We used GEO datasets for NASH and TCGA-LIHA datasets for HCC to screen for hub genes which transform normal liver to NASH to HCC. We found that HMMR was overexpressed in NASH patients compared to health controls, and negatively correlated with poor prognosis in HCC patients, suggesting that HMMR may be a potential monitoring target for prediction of NASH or HCC patients' progression and prognosis.

HMMR acts as an essential component during the polo-like kinase 1 (PLK1)-dependent mitotic spindle positioning pathway, which is required for neural development, neonatal survival, and tumor formation 50. It has been reported that HMMR is overexpressed in numerous tumors, including lung carcinoma, glioblastoma, prostate adenocarcinoma, and leukemia 51-54. HMMR can induce epithelial-mesenchymal transition (EMT) and exert oncogenic effects through activating the TGF-β/Smad2 signaling pathway in gastric cancer 55. Analysis of 1420 colorectal cancer tissues indicates that HMMR may be a more important prognosticator than tumor grade and vascular invasion 56. In this study, we found that HMMR acts as an oncogene activating HCC cell cycle progression and promoting HCC cell proliferation *in vitro* and tumor growth *in vivo*. How HMMR regulates cell cyle-related gene expression remains to be investigated. As far as the regulation of HMMR is concerned, a HMMR antisense lncRNA, HMMR-AS1, can stabilize HMMR mRNA and promotes cancer progression in lung adenocarcinoma, glioblastoma, and epithelial ovarian cancer 57-59. We show that OA stimulates the expression of HMMR through transcription factor CEBPα, providing another mechanism underlying HMMR expression.

Hyaluronan (HA), a member of the glycosaminoglycan family, is an extracellular matrix component and interacts with various cellular receptors to promote cell growth and movement 60. HMMR is one of defined hyaluronan cellular receptors. The evaluation of serum HA could predict liver fibrosis and liver damage in NAFLD patients 61. HMMR was shown to regulate cancer progression in HA-dependent and -independent manners 52, 62. Tissue cells and cancer cells with HMMR overexpression tend to be highly proliferative. The exact role of HA/HMMR axis-mediated NASH/HCC remains to be investigated.

Besides HMMR, the other four hub genes (UBE2T, PTTG1, GINS2 and TYMS) have been shown to play a role in HCC. UBE2T, a member of the E2 family, is demonstrated to be a vital regulator of tumor progression in several cancers, such as lung cancer 63, glioblastoma 64, and HCC 65. PTTG1 has been reported to be associated with poor prognosis in multiple myeloma 66, prostate cancer 67 and HCC 68. Overexpression of UBE2T and PTTG1 promotes HCC cell growth, migration and invasion through activating Akt pathway 69, 70. GINS2 is a member of the GINS complex and participates in DNA replication and cell cycle regulation in most tumors, such as pancreatic cancer 41, bladder cancer 71, and lung cancer 72. Bioinformatic analysis showed that both individual GINS2 and the whole GINS complex could be prognostic biomarkers for HCC 73. TYMS is a rate-limiting enzyme in the nucleotide biosynthetic pathway, and involves in DNA synthesis and repair 40. Interestingly, although upregulated TYMS was associated with poor survival in HCC patients 74, enhanced TYMS activity may protect cancerization of liver tissue by minimizing uracil misincorporation into DNA 75. However, there are no reports on the relationship between these four hub genes and NASH. More research will be needed to understand the association.

## Supplementary Material

Supplementary figures.

Supplementary tables.
