# Supplementary material for: Oleate acid-stimulated HMMR expression by CEBPα is associated with nonalcoholic steatohepatitis and hepatocellular carcinoma
Source: Int J Biol Sci. 2020 Aug 27;16(15):2812–27. doi: 10.7150/ijbs.49785 (PMC7545721; doi:10.7150/ijbs.49785)
Supplement: Supplementary file 1 — Supplementary figures. [file ijbsv16p2812s1.pdf]

Figure S1

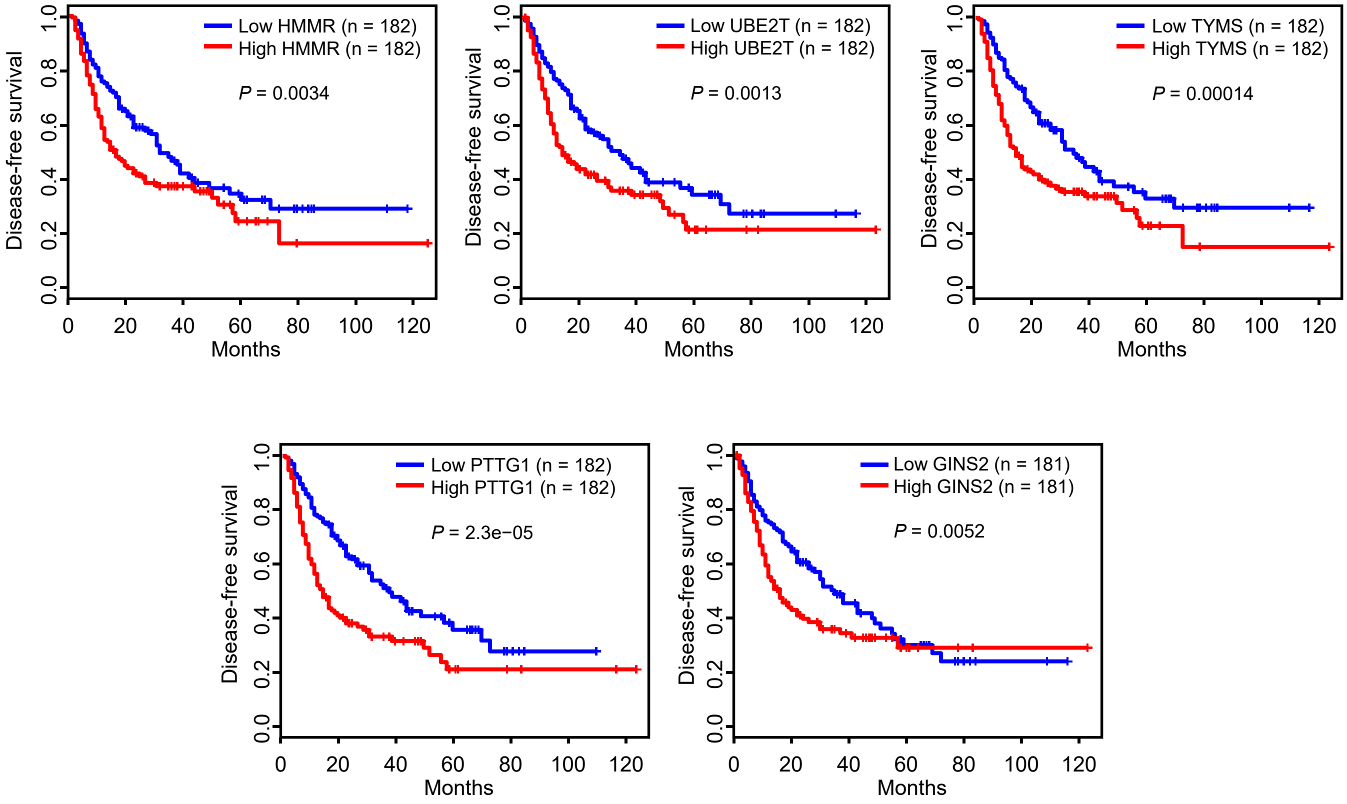

Figure S1. The association between five hub genes and disease-free survival in HCC patients. The correlation of the expression levels of five hub genes, namely HMMR, UBE2T, TYMS, PTTG1, and GINS2, with disease-free survival in HCC patients from TCGA-LIHC dataset was determined.

Figure S2

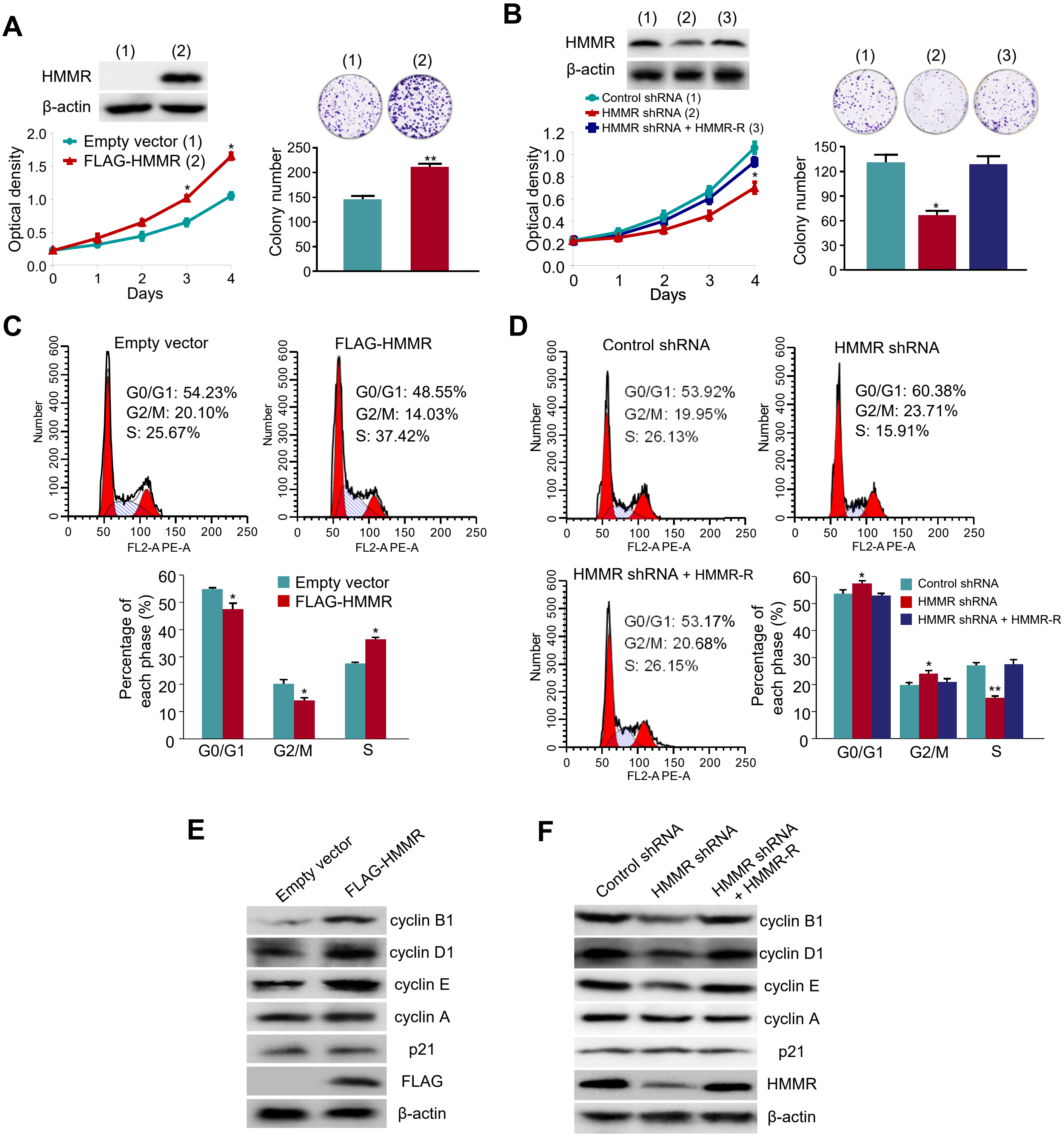

Figure S2. HMMR promotes MHCC-97H cell proliferation through activating the G1/S and G2/M transitions. (A) MHCC-97H cells transfected with FLAG-HMMR or empty vector were grown in regular medium and harvested at the indicated times. Cell number and colony formation were detected. Immunoblot showed the expression of FLAG-HMMR. (B) MHCC-97H cells infected with control shRNA, HMMR shRNA or HMMR shRNA plus HMMR-R were cultured and analyzed as in (A). Immunoblot showed HMMR expression. (C) Flow cytometry analysis of cell cycle in MHCC-97H cells transfected with FLAG-HMMR or empty vector. (D) Flow cytometry analysis of cell cycle in HepG2 cells infected with HMMR shRNA or HMMR shRNA plus HMMR-R. The image displayed is one of the representative results. Data shown are mean ± SD of triplicate measurements that have been repeated 3 times with similar results (A-D) (*P < 0.05, **P < 0.01 versus corresponding control). (E) Representative immunoblot of MHCC-97H cells transfected with FLAG-tagged HMMR or empty vector. (F) Representative immunoblot of MHCC-97H cells infected with control shRNA, HMMR shRNA or HMMR shRNA plus HMMR-R.
