# Supplementary material for: Oleate acid-stimulated HMMR expression by CEBPα is associated with nonalcoholic steatohepatitis and hepatocellular carcinoma
Source: Int J Biol Sci. 2020 Aug 27;16(15):2812–27. doi: 10.7150/ijbs.49785 (PMC7545721; doi:10.7150/ijbs.49785)
Supplement: Supplementary file 2 — Supplementary tables. [file ijbsv16p2812s2.pdf]

Table S1. The cDNA target sequences of shRNAs or siRNAs

Gene	Sequence (5’→3’)
HMMR siRNA-1	GGAGTCTCGAAGAGTCTCA
HMMR siRNA-2	GTTCTAGGTATCAAACACT
HMMR siRNA-3	CAGCTGGAAGATGAAGAAG
CEBPα siRNA-1	CGGTGGACAAGAACAGCAA
CEBPα siRNA-2	GCTGACCAGTGACAATGAC
CEBPα siRNA-3	CCTTCAACGACGAGTTCCT
HMMR shRNA	GTTCTAGGTATCAAACACT

Table S2. Primers used for RT-PCR or ChIP

	Forward(5’→3’)	Reverse(5’→3’)
RT-PCR		
HMMR	ACCAACTCAAGCAACAGGAGGA	CCTGAGCTGCACCATGTTCATT
CEBPα	GCCAAGAAGTCGGTGGACAAGA	GGCGGTCATTGTCACTGGTCAG
β-actin	ATCACCATTGGCAATGAGCG	TTGAAGGTAGTTTCGTGGAT
ChIP		
HMMR-1	CTGCTGTGAGCTACAATCTCA	GTACGTATAGACCAGTGGGATTT
HMMR-2	CATACCAGTGAATACTCCAAG	TGTTTGATGGCAGTGTAATATGG
HMMR-3	CTGTTGTCTTCCTGCCTGTC	GCAGGTGCTGAGTTGTGGAA
HMMR-4	CTAAACTCGCAGGCCAGCTAA	CTACCGGTTTCAAATTCAAATCTCC

Table S3. Characteristics of 50 HCC patients from the TCGA-LIHC dataset

Characteristic	Value
Age (mean ± SD)	61.68±15.96
Sex (male/female)	28/22
BMI	25.14±7.69
Pathologic_M (M1/M0/NA)	1/33/16
Pathologic_N (N2/N1/N0)	1/31/18
Pathologic_T (T4/T3/T2/T1/NA)	3/13/14/20
Stage (IV/III/II/I/NA)	1/12/11/18/8
Ethnicity (white/black/ASIAN/NA)	34/7/6/3
Survival status (dead/alive)	34/16

Table S4. The statistical results of the connectivity of the network

Node name	LOG_{10}(MCC)	DMNC	MNC	Degree	EPC	Expressed in NASH/HCC
HMMR	13.96	0.985	19	19	16.559	Up
UBE2T	13.96	0.9099	24	24	17.751	Up
TYMS	13.96	1.04399	20	20	16.871	Up
PTTG1	13.96	0.97661	22	22	17.469	Up
GINS2	13.96	1.02307	21	21	17.365	Up
